# Adherence to Antihypertensive Medication: An Interview Analysis of Southwest Ugandan Patients’ Perspectives

**DOI:** 10.5334/aogh.2904

**Published:** 2020-06-10

**Authors:** Josephine Nambi Najjuma, Laura Brennaman, Rose C. Nabirye, Frank Ssedyabane, Samuel Maling, Francis Bajunirwe, Rose Muhindo

**Affiliations:** 1 Department of Nursing, Mbarara University of Science and Technology, Mbarara, UG; 2 Ron and Kathy Assaf College of Nursing, Nova Southeastern University, Colonial Court, Fort Myers, Florida, US; 3 Department of Nursing, Makerere University, Kampala, UG; 4 Department of Medical Lab Science, Mbarara University of Science and Technology, Mbarara, UG; 5 Department of Psychiatry, Mbarara University of Science and Technology, Mbarara, UG; 6 Department of Community Health, Mbarara University of Science and Technology, Mbarara, UG; 7 Department of Internal Medicine, Mbarara University of Science and Technology, Mbarara, UG

## Abstract

**Background:**

Hypertension is a significant cardiovascular disease (CVD) and driver to CVD disorders in sub-Saharan Africa. It is a major independent risk factor for heart failure, stroke, and kidney failure. Persons living with hypertension attend to many aspects of self-care to manage their condition, including high blood pressure medication adherence to control of blood pressure. Rates of medication non-adherence, and thus uncontrolled hypertension, remain high and contribute to poor health outcomes. Understanding barriers and facilitators to adherence to hypertension therapies can help improve health outcomes.

**Objective:**

The aim of the study was to describe the common reasons for adherence and non-adherence to antihypertensive medication from patients’ perspectives.

**Methods:**

A qualitative study engaged clients of an out-patient clinic of a regional referral hospital in southwestern Uganda who were living with hypertension as participants. One-on-one in-depth interviews provided the narrative data. The interview transcripts were analyzed using thematic analysis.

**Findings:**

Sixteen participants provided the data for the findings. The themes identified as facilitators for adherence to antihypertensive medication were patients’ understanding of prescribed medication, availability of medication for hypertension, family support for patients living with hypertension, and regular review appointments at the hypertensive clinics. Conversely, lack of supply in government dispensaries, use of self-prescribed analgesic medication, and stigma were identified as barriers and challenges of adherence to antihypertensive medication.

**Conclusions:**

There is an urgent need for the health ministry to improve availability of high blood pressure medication and for health care providers to deliver individualized patient centered care, and sensitization on danger of self-prescription and measures that reduce stigma. These strategies may improve adherence to high blood pressure medication.

Cardiovascular disease is a leading cause of death in Africa after HIV [1,2]. Hypertension is a significant risk factor for developing life-altering or life-ending cardiovascular diseases [2,3,4,5] and is the most important preventable cause of heart disease and stroke worldwide [6]. In 2010, the global burden of disease study reported that hypertension was found to be the leading risk factor for morbidity globally [7]. Worldwide, hypertension is a serious public health challenge [8], and in developing countries it poses a more severe threat to individuals’ wellbeing [9,10]. Despite hypertension being the most common noncommunicable disease (NCD) in Uganda [11], in sub-Saharan Africa (SSA), health prevention and treatment initiatives remain primarily focused on communicable diseases like tuberculosis, HIV, and malaria. Failure to diagnose and control hypertension is a major cause of cardiovascular morbidity and mortality in Africa [1,12]. Although improvement in detection and treatment of hypertension methods have been designed and implemented worldwide, poorly managed hypertension and its complications remain a major cause of morbidity and mortality in SSA.

Medication adherence is crucial for successful treatment of chronic conditions like hypertension and diabetes. Consistent adherence to antihypertensive medication is strongly associated with effective blood pressure control [13]. Non-adherence is a widespread problem, and the action can be intentional or non-intentional [14]. Researchers who examined this issue in 12 African countries reported high frequency of poor adherence among patients with hypertension [15]. These findings support the understanding that non-adherence is significant public health problem contributing to increased mortality, morbidity, and to excessive health care expenditure while decreasing quality of life for the population [16].

Although antihypertensive medication adherence is important for positive health outcomes [17,18], studies report low adherence rates [12]. In Kampala, Uganda, 17% of stroke survivors reported adherence to their prescribed antihypertensive medications; researchers cite lack of knowledge as the main contributor to the poor adherence [19]. Despite medication adherence being key area of interest among practitioners and researchers, a gold standard intervention does not exist [16]. The aim of this study was to understand from participants’ perspectives what factors create challenges and barriers to hypertension treatment adherence and what factors facilitate treatment adherence. Findings from this study will ultimately inform nurse-led patient-centered interventions to improve adherence to hypertension therapies.

## Materials and Methods

### Design and Setting

The study sample and data for this qualitative study were obtained at an outpatient clinic at Mbarara Regional Referral Hospital in southwestern Uganda. The physician-run clinic is staffed by a physician or a resident, or a medical officer assisted by a diploma nurse. The clinic is open one day each week for outpatient management of hypertension.

Individuals with hypertension come to this government health facility at no cost. The service is available once a week on clinic day (Tuesday). The clinician prescribes antihypertensive medication that the health facility dispenses to the patients without cost. However, stock-out of most of these medications occur regularly. During the stock-out, patients who can afford, may buy antihypertensive medication from nearby privately owned pharmacies. Patients without the financial resources to procure privately supplied medications go without.

The proposed Uganda National Health Insurance Scheme [20] was approved in 2019, but not yet implemented [21]. Individuals with higher paid jobs in the corporate sector may purchase private coverage or receive it as a benefit from their employers. Lower cost micro-insurance schemes exist in Uganda [21], but they do not cover chronic conditions like hypertension. Generally, individuals who use the government health services must incur the cost of anti-hypertension medication directly and individually when the stock-outs occur.

### Ethics Approval and Consent to Participate

The study was reviewed and approved by Mbarara University of Science and Technology Research Ethics Committee (MUST REC -17/11-17) and the Uganda National Council for Science and Technology (UNCST) (HS 2389). Participants gave written informed consent for participation and audio recording of the interviews, which occurred in private rooms.

### Sampling and Recruitment

Hypertension clinic clients were eligible for inclusion as study participants if they had been diagnosed with hypertension for a minimum of six months and lived within a 30km radius from the hospital. Acutely ill clients and those with hearing or speech problems or who had a clinical diagnosis of severe persistent mental illnesses were excluded. The research assistants (RA), were nurses not affiliated with the clinic. They approached clients identified by clinic workers as meeting inclusion criteria after clients completed their interaction with the clinic provider.

### Data Collection

Data were collected via one-on-one in-depth interviews with participants. We used an interview guide based on literature about medication adherence to aid data collection. It included questions about factors that influence adherence, intentional and non-intentional causes of non-adherence, the knowledge about prescribed medications, the anticipated effects of the prescribed medications, the lived experience while taking antihypertensive medication, and ways in which each participant adapted to improve medication adherence [22,23,24]. The interview guide was developed in English by the research team (interview guide in supplemental material). The research assistants, who are fluent in Runyankole/Rukiga (commonest language in western Uganda) and experienced in translation to Runyankole/Rukiga, translated the interview guide. A professional translator translated it back to English to check for translational meaning, accuracy, and conceptual equivalence. The final refined Runyankole/Rukiga interview guide was used to conduct the interviews after a consensus meeting between the two translators and the principle investigator.

Two research assistants shared the data collection process during the interviews. One research assistant, a graduate nurse and experienced interviewer, conducted the one-on-one interviews. The other research assistant was the notetaker/transcriber who also operated the audio-recorders during the interview. After the interview, the notetaker, shared the notes with the principal investigator and transcribed the recording in Runyankole/Rukiga. After every three interviews the following translation and cross check process occurred before the next set of interviews. First, the Runyankole/Rukiga transcripts were translated to English by a professional translator; second, the interviewer compared the final two transcripts to cross-check for meaning and completeness; and third, the principal investigator reviewed the content and briefed the interviewer and notetaker about the conduct and interview guide content. The interviews lasted from 30 to 50 minutes. We achieved data saturation after interviewing 16 participants.

### Data Analysis

Data analysis was an interactive process starting from the first transcripts and continued throughout the data collection. Two members of the research team read the English version of the of the transcripts to gain familiarity with the data before development of codes [25].

The transcripts were analyzed using thematic analysis. The researcher read the transcripts repeatedly, wrote notes in the margins that we developed into codes, and merged into themes. Description of the codes under every theme is presented and their connection to participants’ medication adherence. A codebook for analysis was developed and agreed upon by coinvestigators. Data were re-analyzed with the aid of Nvivo 12.2.0.443 Pro [26]. More subthemes and themes emerged from the data after entering it in Nvivo. Subthemes and themes were shared with the interviewer and notetaker to ensure that they contained all the issues expressed during the interviews. Results are divided into broad categories, factors that facilitate antihypertensive medication adherence and challenges or barriers that impede adherence. In the result section, representative verbatim quotes are referred to by the participant’s study number (1–16) and age range.

## Results

### Study Respondents

The sixteen participants’ ages ranged from 23 to 85 years (*m* = 51.7, *SD* = 17.8) with a mean number of years of living with a confirmed hypertension diagnosis of 5.8 (range of 1 to 40). Only one participant stayed at home alone. Twelve participants were subsistent farmers, two were involved in small scale businesses, two were employed, one accountant, and one teacher. Seven were married, six were widowed, and three were divorced (Table 1).

**Table 1:** Study participants.

Item		*M (SD)*

Participants age		51.78 (17.84)
Monthly income (USD)		56.51 (63.45)
Time to travel to the clinic (hours)		1.32 (1.14)
Cost to travel to the clinic (USD)		2.03 (1.77)
Time of hypertension diagnosis (years)		6.00 (9.63)
Number of household members		3.69 (2.36)
		***n* (%)**
Level of Education	No formal education	5 (31.25%)
	Completed Primary education	3(18.75%)
	Not completed primary	5 (31.25%)
	Completed Ordinary level	2 (12.5%)
	Completed University	1 (6.25%)
Mode of Accessing the clinic	Walking	1 (6.25%)
	Motorcycle for hire (Bodaboda)	7 (43.75%)
	Public vehicle	6 (37.5%)
	Public vehicle + Bodaboda	2 (12.5)
Occupation	Subsistence farmers	12 (75%)
	Small Scale Business	2 (12.5%)
	Formal Employment	2 (12.5%)

Exchange rate 1USD = UGX 3600.

## Medication Adherence Facilitators

### Family Support for Patients with Hypertension

Most participants reported that family support was important in the management of hypertension. This support was mainly from their children and other immediate relatives. The assistance from these family members compelled them to adhere to antihypertensive medication. Family support was defined as support given to patients with hypertension by a family member or a close relative. This ranged from physical support to financial support. Family support took the form of reminders to take the antihypertension medication, advice about reasons to take the medication, provision of access to health care through transportation, and funds to buy the medication. According to participants who reside in a home with a family member, irrespective of the age, being reminded to take their medication was important. This is illustrated in the following quotes below:

“Mum always reminds me, actually when we finish taking breakfast, she asks me whether I have taken medicine. At times I tend to forget, and she tells me to have breakfast and take medicine.” (Participant 10, 30–40 years)

When household members knew that the participant suffered from hypertension, they mentioned that they helped remind them take medication. Children of all ages were also important in reminding and guiding participants and/or their parents/guardians to take their medication:

“My daughters are the ones who remind me. They remind me that ‘mummy, have you taken your medicine? Take the medicine so that, mummy, you won’t die.’” (Participant 15, 30–40 years)

Participants with older children had more advantage. The children not only reminded their parents to take their medication, but they also helped with access to care. Participants reported that their children took them to the hospital for follow-up visits when possible:

“I go to the clinic because my son has a vehicle, he brings me here and I get it [antihypertensive medication].” (Participant 7, 30–40 years)

In some cases, where the participants did not access medication at the public dispensary, and/or they could not afford to buy medication for themselves, their immediate family members helped to buy the medication from private pharmacies. This was affirmed by some of the participants in the study:

“I used to buy it in our trading center, but failed. I talked to my brother, who buys it in the city and sends it by bus and my sisters, who are studying in town would pick it for me. When I come from home to town for review, I find it in town. But still I must be having some.” (Participant 14, 40–50 years)

“My husband is a driver. Most of the time [he] is on journey; he does not help me a lot, but he helps me when there’s no medicine in the hospital. He buys it from the clinic.” (Participant 15, 30–40 years)

“When I get someone who helps me like among my children, he asks for the book and brings the medicine. That’s when I get the whole dose that was prescribed for me.” (Participant 6, 60–70 years)

Most participants describe the relationship of their condition as dependent to their family wellness. This was coupled with home routines like radio and television station news bulletins.

### Availability of Antihypertensive Medication

Antihypertension medication is prescribed to be taken daily. Medication availability was highlighted as a compelling factor for most participants. Participants reported when the medication was available to the participants, they would take it. These participants sometimes receive medication from the clinic dispensary at no cost (this is an arrangement by the government), or because all the prescribed medication might not be available at the clinic dispensary, half of the prescription may be dispensed depending on the availability. The medication at the clinic dispensary is free; whereas, if patients have to get it from elsewhere, they will have to pay for it. As reported by one of the participants:

“At first, I used to go, and they give me two types of drugs, and they would tell me to go and buy the third type. I buy it from town.” (Participant 4, 70–80 years)

In other instances, participants get medication from colleagues and friends. One participant, who was taking amlodipine, reported having a neighbor who worked in a different clinic that had the medication in stock. The neighbor supplied approximately 100 pills to the participant. Sometimes the doctors prescribed medication using brand names that participants could not afford or were costly and participants could not afford the prescribed dose. Because the participants had to buy medication, the availability of such medication eventually affected adherence:

“I don’t want to deceive you, when I don’t have money I miss. If I have money, because a tablet is 1,500 (UGX) = 0.42USD, I go and buy from town and take. Or at times, I don’t buy it in time, and I take it like at night. There you find you’re skipping. Let’s say you’re taking in the morning; you go and buy it in the evening and take it at night. So, there you see there’s a problem. There’s when you don’t have money, then when you get, you can buy a dose for some months. Like now I have for 2 months.” (Participant 10, 30–40 years)

Participants reported that when medications were available, their chances of taking the medications increased, although the cost has greatly affected the availability and hence, adherence:

“…there’s when you wake up with too much headache. At times you don’t have money to buy medicine, because for us we don’t get this medicine from here; I last got medicine from here in 2014 and ever since I’ve been buying. So, there’s when I don’t have money for buying, I stop a bit, after stopping, goes very high. [I: mm] [short pause] At times you feel tired, sometimes…” (Participant 10, 30–40 years)

### Understanding the Prescribed Medication

Participants described lack of understanding why medications were prescribed and what each medication was intended to accomplish. In most cases, participants reported at the review clinic with another presenting complaint in addition to their hypertension. These included complaints related to hypertension (complications and medication side effects) and those unrelated to hypertension, for example, headache and peptic ulcers. The doctor at the clinic prescribed medication for all the presenting complaints. In the end, participants adhered to medications that were accessible or that had mild or no side effects. The participants did not necessarily adhere to the most important medication(s) to manage their hypertension. Participants felt compelled to adhere to the medication if they understood the dosing, reason for the prescription, the expected outcomes, potential side effects, and the duration for taking the medication.

Some patients not only misinterpreted the prescription on their own, but also were not able to read what was written for them at the dispensary by the pharmacists. Thus, they did not know what to do with the pills that were given to them. One of the participants reported that they could wait for the family member to interpret for them:

“Though I’m not educated, when I get medicine, I tell my children to read for me the prescription and guides on how to take.” (Participant 12, 40–50 years)

One participant who had been taking antihypertensive medications for three years mentioned how she was helped by the doctor after using the medication inappropriately for a long time. She reported that:

“…there’s a time I found a young man and he told me, ‘old woman, you don’t take your drugs right.’ He said, for example, you’re given drugs here and they tell you two times one, so let’s say, for example, you might find that in the morning I have two types of drugs and at night two types. So, for me when I see that one of them has reduced, what I was taking in the morning is no longer there, I change the time for the other one… start taking it in the morning, because, I’m like how will I go through the day? He told me, you’re misusing the drug. If you decide to take it at ten, make sure it’s ten; take all the drugs at ten. Then you will find that this high blood pressure has done what, and that’s why you see some time ago my pressure was found to be 130. I think it’s because I followed that young man’s instructions…” (Participant 9, 60–70 years)

In addition, when patients did not understand the prescription, they tended to abruptly stop taking the medication. When they felt better, they just stopped taking antihypertensive medication. Some participants, when the first prescription was finished, they just stopped taking the medications:

“…But there’s when I spend like a week without taking medicine, because sometimes I feel I’m okay, then I leave. But there’s time when I feel I’m weak, when I’m attacked. I feel pain in my arm, then I realize that it’s pressure; I take the medicine and feel fine…” (Participant 11, 30–40 years)

Participants who did not stop abruptly felt the review clinic appointment was not necessary and they refilled their pills without doctor’s guidance. Some participants mentioned that they just took the previous prescription to the pharmacy and refilled the pills sometimes.

**Figure 1:**
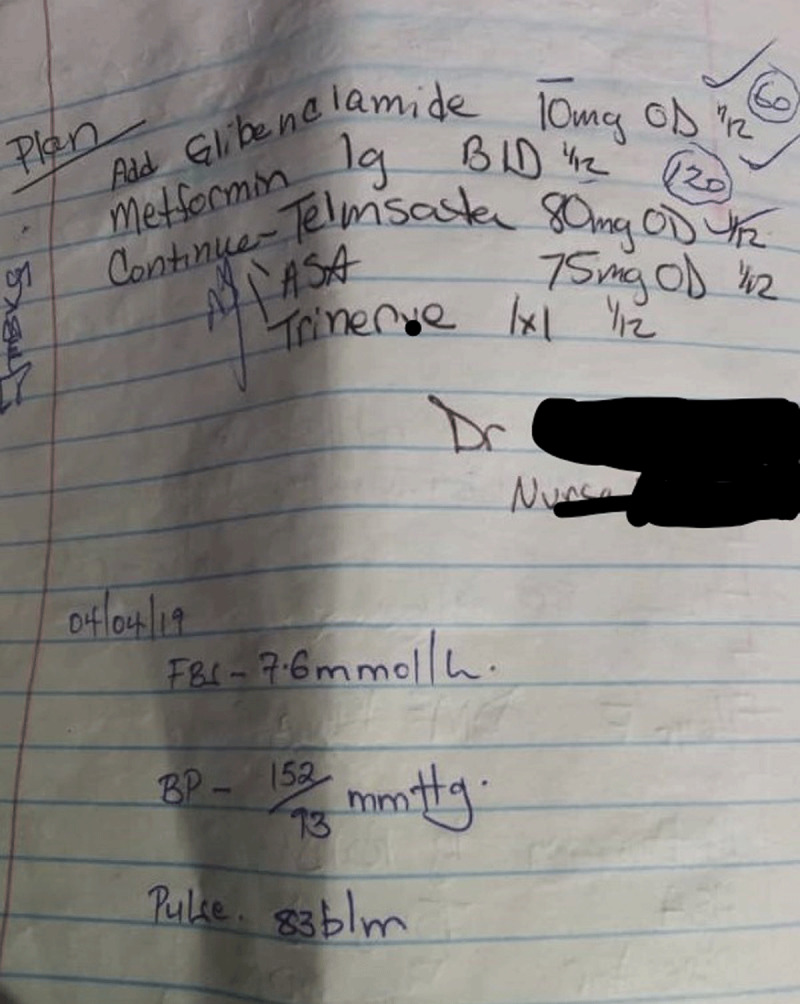
Sample review and medication page from the patient with hypertension notebook.

**Figure 2:**
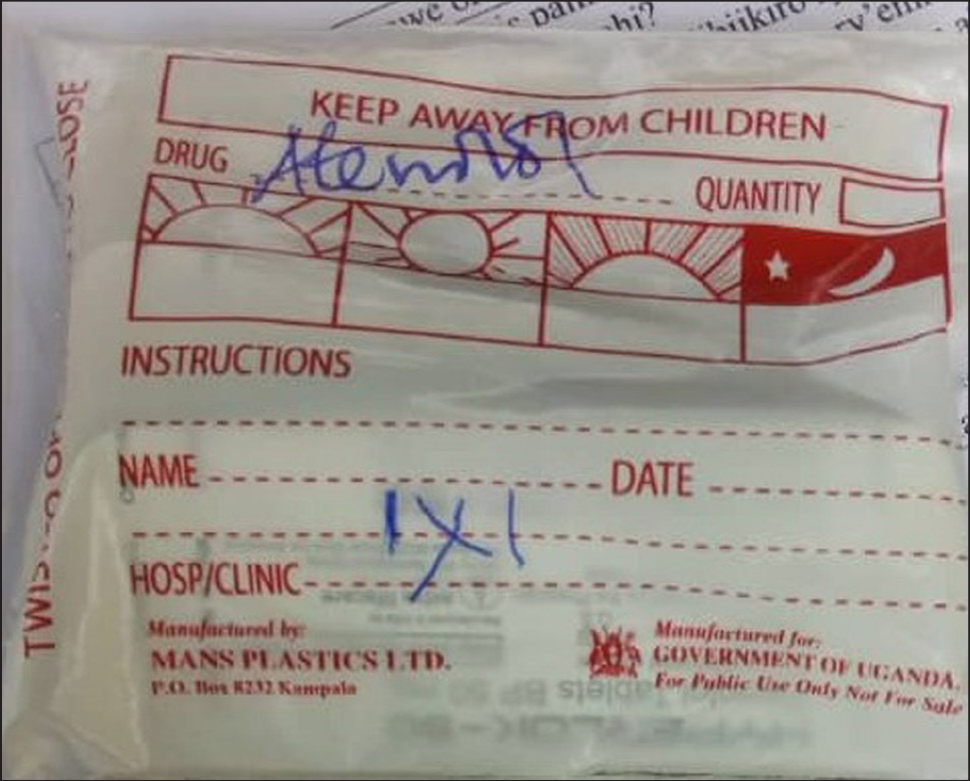
Sample pharmacy label of Atenolol for a patient with hypertension from public/government pharmacy.

**Figure 3:**
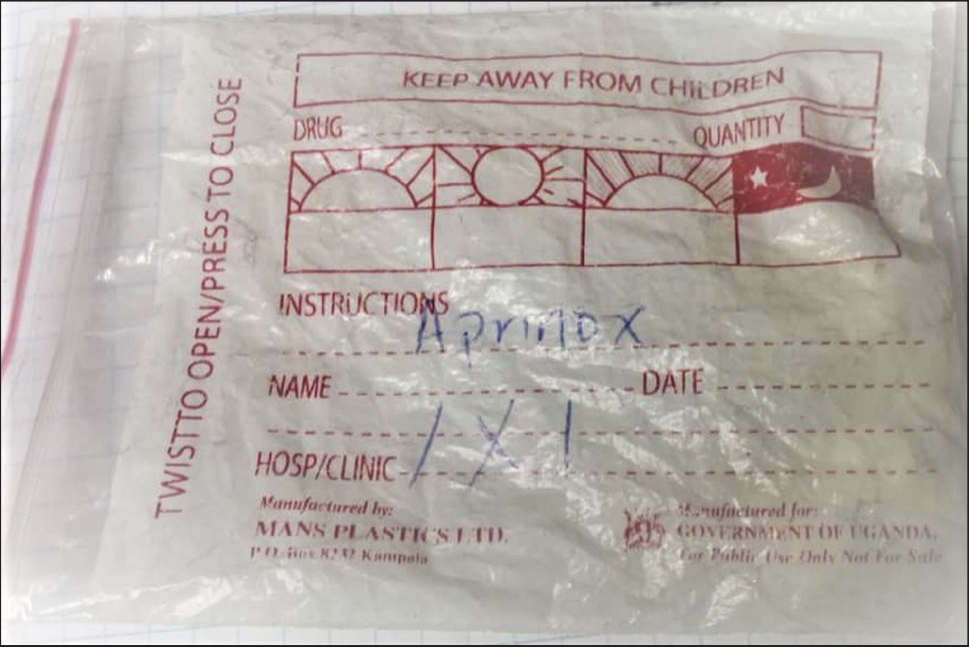
Sample label of Aprinox given to a patient with hypertension.

**Figure 4:**
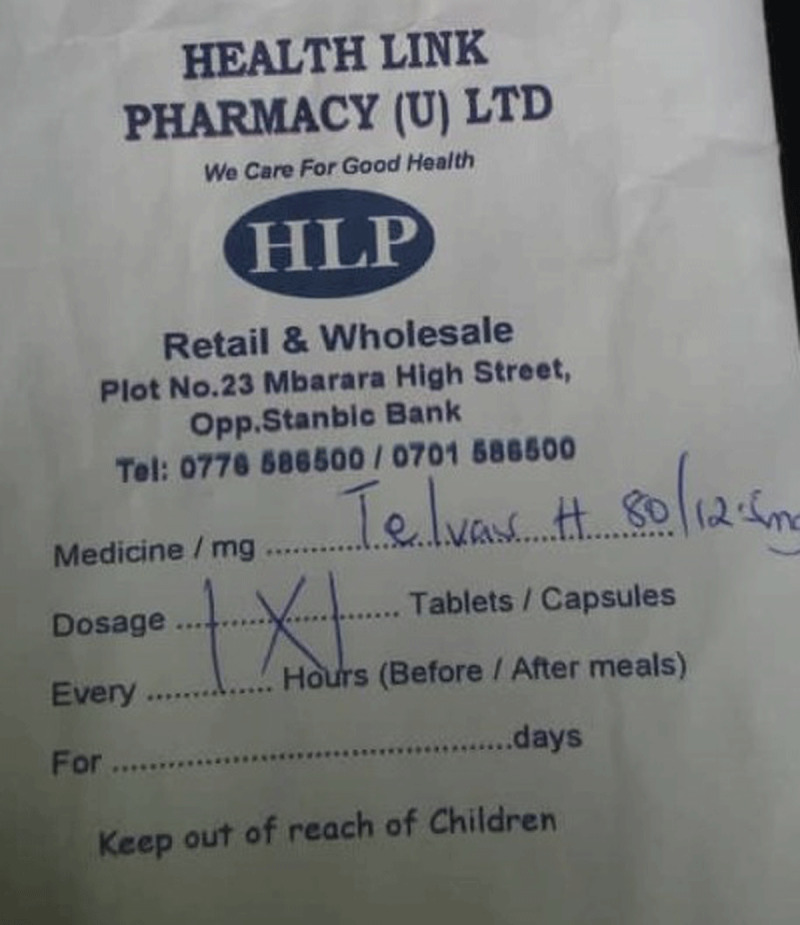
Sample label of Telvas H given to a patient with hypertension from a private pharmacy.

For every visit, the patient carries a small notebook to the review clinic where the doctor and/or nurse writes the review notes (vital sign measurements, recommendations, and prognosis) and prescribed medication(s) for that visit. The patient uses the book to access the medication at either the public dispensary or when he/she takes the written prescription to the private pharmacy to buy the medication. Participants reported notes written in the book were illegible (See Figure 1). At the dispensary, the prescriptions were not explained to the participant and instructions written on the envelope containing the medication were not self-explanatory (see Figures 2, 3, and 4). The participants did not take the medication as prescribed, or they started taking according to instructions when a family member who understood the numbers written on the envelope or box was available to interpret them to the participant.

## Barriers/Challenges to Adherence to Antihypertensive Medication

### Unavailability of Unaffordability of Prescribed Medications

Lack of medication availability diminished the participants’ adherence. Some participants could not afford the cost of buying medications, or they decided not to buy it. In these situations, adherence occurred only to medications that were available:

“When you find that the medicine is not here [Clinic dispensary], sometimes they don’t buy them, because I also used to do it. When I would leave here, I would ignore. When I have only one dose, I remain with only that.” (Participant 10, 30–40 years)

Most of the participants who were prescribed more than one pill for high blood pressure control reported taking only the pills they could afford. Some participants who could afford buying all prescribed pills did not take the pills that gave them side effects; this was not discussed with the prescribing physician:

“… I don’t know whether it’s the pressure that brings the kidney or the kidney that brings pressure. I… You don’t know? [shakes his head] only that I do all the tests and they give medicine but I don’t know how it works. I don’t know how it is, but I see I’m not helped.” (Participant 1, 20–30 years)

Participants reported they get pills that are available in the public clinic dispensary and buy medications that are not available from private pharmacies/drug shops. In some cases, they will take only the available medication, or they buy fewer pills than the prescribed dose, up to what they can afford.

### Use of Self-prescribed Painkillers

Because most of the participants reported being in some sort of pain at some point, it was common that participants sometimes took analgesic medications. Participants mentioned they took pills (analgesics) that were not prescribed by the doctor. Participants bought the analgesics at private pharmacies and drug dispensaries:

“I have been taking this tablet once a day for general pain for the last two years [shows indomethacin tablets to interviewer]. Before, I was taking diclofenac for about three years and I was changed. I take it whenever I feel pain. When my medication is over, I just buy others from the drug shop.” (Participant 3, 50–60 years)

The reasons for taking these pills is not clear, although the researchers assumed that the participants were treating pain due to a complication of hypertension or due to the antihypertensive medication side effect. This is because some participants took the pills when they were not feeling well or when they did not take their antihypertensive medication. This pattern implied that they either took the medication to solve pain as a side effect for the drug or as a hypertension complication.

### Missing the Clinic Follow-up Appointment for Hypertension Review

At the conclusion of each clinic visit the participants were given a clinic review date depending on the severity of their condition and medication given. Most of the participants who were able to refill their pills did not come to the clinic until they had a complication. Other participants reported that they could only come back after the quantity of pills in the prescription were finished, even if they got side effects related to the antihypertensive medications. Participant had tendency to wait for the proposed review date, even when they did not feel well.

### Stigma Associated with Taking Antihypertensive Medication Daily

According to participants, the public perception of taking medication daily was associated with treatment HIV with anti-retroviral medications (ARV). This association created stigma for taking daily medications. Some participants would not take medications to avoid being seen by family and significant others to prevent the perception of taking ARV for HIV. They reported that after being seen by other people taking medication every day, others would conclude that the participant is using hypertension as a cover, while really taking ARVs, when this is not the true situation:

“If you’re taking medicine every day, you feel as if you’re not like others. Because, you ask yourself why you’re on medicine while others are not taking! So, you appear as if you’re abnormal. Sometimes people start criticizing you, saying that maybe you have HIV, but you’re hiding it, or maybe I did something wrong. So sometimes you hate yourself. Why are you taking drugs [snaps her fingers] all the time?” (Participant 10, 30–40 years)

Participants expressed that when telling their family members that they experienced complications like palpitation, sweats, and shivers, their immediate significant others did not believe that this was due to hypertension but thought it was due to other diseases.

## Discussion

The study shows different interconnected factors that affect hypertension medication and treatment adherence among patients in southwestern Uganda. According to most study participants, there was a strong relationship between availability of medication and good adherence. Lack of availability of medication at the government dispensary translated directly to the increased costs of the antihypertensive medication and was inversely related to medication adherence. Our results are the similar to those of earlier studies in low-resource settings that describe how participants who were able to buy or access antihypertensive medication reported higher adherence to medications [2,19]. A quantitative study conducted a few years earlier in the same district revealed that participants who bought antihypertensive medication from a private pharmacy and participants who had family members buy antihypertensive medication demonstrated higher medication adherence rates [22]. Multiple studies have reported that lack of medication at Uganda government dispensaries creates a challenge to management of hypertension [12,19,27]. The same situation has been reported for patients with hypertension in Tanzania, where high medications costs correlated with decreased medication adherence [13].

Participants’ understandings of the reason for the prescribed treatment, their diagnosis, and the relationship between treatment and prevention of complications was a powerful and compelling factor that promoted adherence to medication. Participants who reported not understanding the reason for the medication or their diagnosis had a tendency of abruptly discontinuing the medication. Participants from this sample and in prior studies who understood how to take the prescription adhered to the antihypertensive medication [19]. Researchers reported that good knowledge of hypertension was associated with better hypertension control through adherence among adult patients with hypertension in Tanzania [13]. Lack of knowledge contributing to non-adherence is not unique to SSA; a study from Saudi Arabia, showed that only 38.6% of the participants understood the prescription of the commonly prescribed drugs [28]. This finding in our Uganda study is a common challenge worldwide, but has not been largely studied in Uganda.

When participants felt better while taking medication, they reported that they just stopped medication. This was linked to attending doctors’ appointments and understanding about the prescribed medications. Similar observations were seen in Central Manchester, where researchers concluded when patients do not have debilitating symptoms, or anything to compel them to take their medication, they were non-adherent [29,30]. Our participants illustrate how essential it is for health workers to ensure patients understand the current situation, the potential benefits and risks, and the activities required from them while managing their hypertension.

Our participants not only stopped taking medications when they did not have debilitating symptoms, they also stopped keeping follow-up visit appointments. Participants revealed the lack of continued adherence related to lack of understanding the diagnosis. At the follow up visits that did occur, participants noted that lack of medication reconciliation during the visit might have contributed to patients’ use of unprescribed painkillers. Participants reported they got relief when they used certain pills (analgesics) although taking these non-prescribed medications hindered their adherence to the antihypertensive pills. In some cases, participants took analgesics and other medications to treat comorbidities or hypertension complications like renal disease. Participants with other comorbidities that result in chronic pain did not adhere antihypertensive medication. This was also reported in a meta-analysis that included studies from Uganda, where one-third of the patients with comorbidities were not adherent to their antihypertensive medication [31].

Participants’ family support contributed to medication adherence and to follow-up visit adherence. These supportive family members gave help for buying the participants’ antihypertensive medication, which enhanced availability. Prior literature from studies in Uganda [22] and around the world confirm that family is an essential component to therapeutic adherence. Meta-analysis has verified that having interventions tailored to family engagement may improve antihypertensive medication adherence [31].

Participants reported that they sometimes missed medication when they were away from home or being observed by new individuals. Stigma regarding taking daily medication affects adherence; this has been reported regarding antihypertensive, anti-tuberculosis medication, medications for mental illnesses, and antiretroviral medication elsewhere in world and in Uganda [32,33,34,35,36].

### Limitations of the Study

All research has limitations that should be considered when interpreting results. Some of this study’s limitations include that participants might have had more vivid recall because they had just seen the doctor at the time of the interview. Although level of education was associated with understanding of the prescription, we were unable to study such an association. This study was conducted in a single setting in southwest Uganda; the perceptions of this small sample may not reflect experiences of people with hypertension in other settings.

### Policy, Clinical, and Research Implications

**Figure 5:**
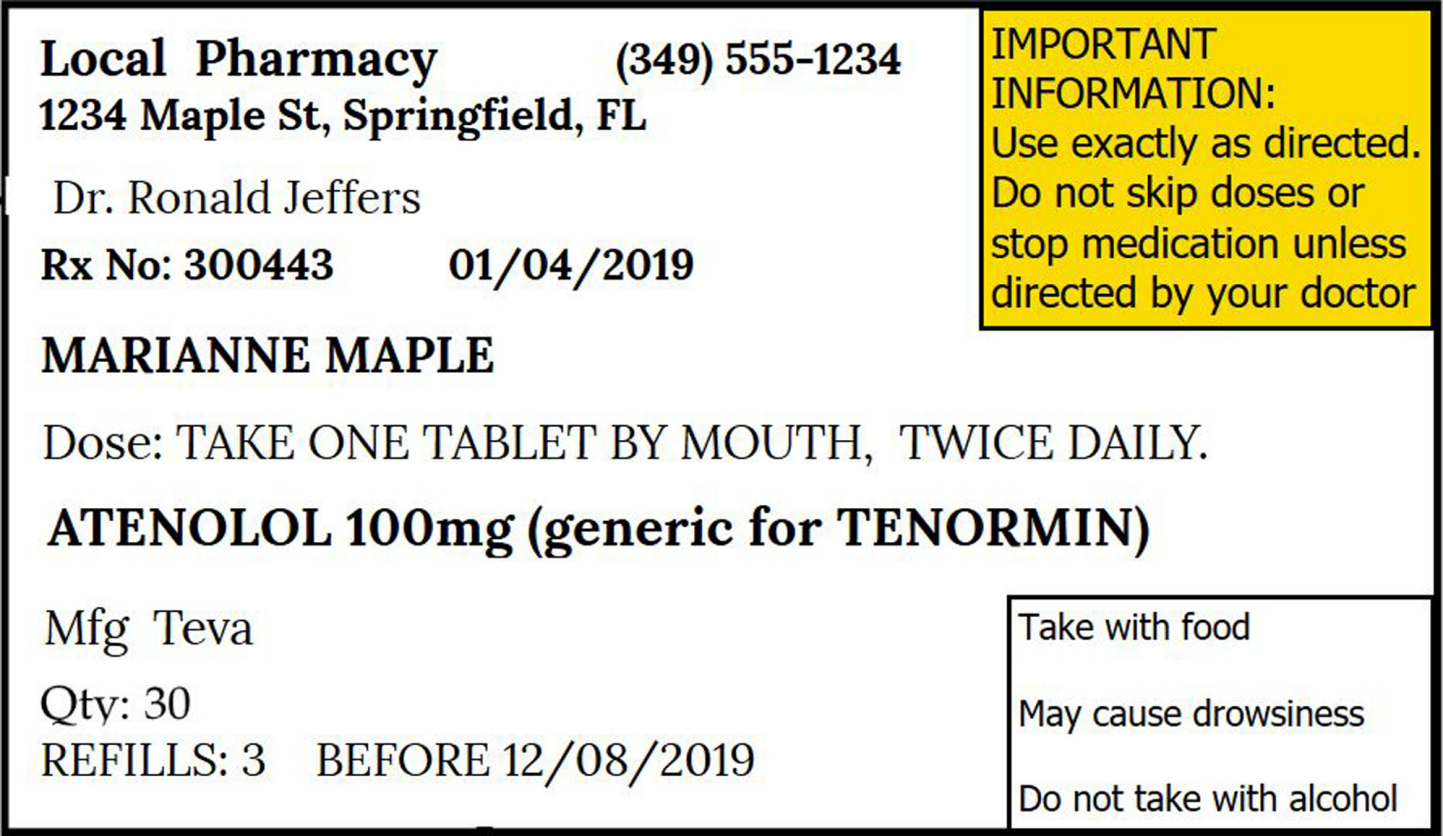
Sample label of Atenolol with recommended components (The dose of atenolol is intentionally higher than normal).

Because the lack of antihypertensive medication availability at the government dispensary was a common thread in the participant interviews, the health ministry should address the supply chain and management of stocks for medications needed to treat NCDs. We recommend that heath workers collaborate closely with their patients to understand whether they are intentionally or not intentionally non-adherent to their prescribed therapies. Health workers should also involve the family members in their patients’ care as these important social strengths help provide critical support. Health care providers should carry out medication reconciliation at review clinics and devise ways to help patients of all levels of education to understand their medication prescription using the available resources. Research should seek low-cost simple interventions by clinic health workers to improve adherence. One example would utilize the patient notebook our study participants described. The patient notebook is currently employed as a communication method between physician, nurse, and pharmacist. Future studies should examine inclusion of patient teaching information in the notebook written by health care workers directed at patients to determine if the existing notebook could be used as a tool to improve medication adherence. The cryptic dosing information written by pharmacists (Figures 2,3, and 4) to instruct patients how to take their medications may not add to adherence by patients as compared to the recommended medication label seen in developed countries (see Figure 5). Using an illustrated label (PictureRx) that depicts the time of day with number of pills is an additional tactic that has improved medication understanding and adherence in low literacy populations and valuable in this low-income multi-lingual country [37]. A study about the potential effectiveness of plain language medication labeling or picture labeling on medication adherence could identify better practices to facilitate medication adherence.

## Conclusions

Factors that influence adherence to antihypertensive medication include availability of antihypertensive medication, patients understanding of the prescription and expected outcomes, and family support for patients with hypertension. Health care providers should leverage these strengths to assist the individuals living with hypertension to adhere to antihypertensive medication. Health care providers should carry out medication reconciliation at the review clinics and emphasize the dangers of using self-prescribed pain killers among patients with hypertension. Encouraging patients to attend the review clinics or return to the hospital whenever they have side effects or complications may enhance adherence. This may be achieved through patient centered communication using the patients’ notebooks as communication tools between the doctors, patients, and places where they get medication. There is need for additional and more effective patient education.

## Data Accessibility Statements

The datasets generated or analysed during the current study are not included in this article but only available once reasonably requested from the corresponding author.

## Abbreviations

CVD: Cardiovascular Disease

HIV: Human Immunodeficiency Virus

ARV: Antiretroviral drugs

NCD: Noncommunicable Diseases

SSA: sub-Saharan Africa

TB: Tuberculosis

UNCST: Uganda National Council of Science and Technology

MUST: Mbarara University of Science and Technology

## Author Information

NNJ is an assistant lecturer in the Department of Nursing and the coordinator of the clinical skills labs and simulation center at the faculty of Medicine MUST LB is an assistant professor of nursing at the Ron and Kathy Assaf College of Nursing, Nova Southeastern University, Florida, USA. RCN is the senior lecturer at the department of nursing at Makerere University; FB is a senior Lecturer in the department of public health MUST, FS is a lecturer in the department of Medical Laboratory science at MUST; SM is a an associate professor of psychiatry at MUST; RM is a physician and a lecturer in the department of internal medicine MUST.
